# Hyperlipidemia and hepatitis in liver-specific CREB3L3 knockout mice generated using a one-step CRISPR/Cas9 system

**DOI:** 10.1038/srep27857

**Published:** 2016-06-13

**Authors:** Yoshimi Nakagawa, Fusaka Oikawa, Seiya Mizuno, Hiroshi Ohno, Yuka Yagishita, Aoi Satoh, Yoshinori Osaki, Kenta Takei, Takuya Kikuchi, Song-iee Han, Takashi Matsuzaka, Hitoshi Iwasaki, Kazuto Kobayashi, Shigeru Yatoh, Naoya Yahagi, Masaaki Isaka, Hiroaki Suzuki, Hirohito Sone, Satoru Takahashi, Nobuhiro Yamada, Hitoshi Shimano

**Affiliations:** 1 Department of Internal Medicine (Endocrinology and Metabolism), Faculty of Medicine, University of Tsukuba, Tsukuba, Ibaraki 305-8575, JAPAN; 2 International Institute for Integrative Sleep Medicine (WPI-IIIS), University of Tsukuba, Tsukuba, Ibaraki 305-8575, Japan; 3 Laboratory Animal Resource Center, University of Tsukuba, Tsukuba, Ibaraki 305-8575, Japan; 4 Department of Hematology, Endocrinology and Metabolism, Niigata University Faculty of Medicine, Niigata, Niigata 951-8510, Japan; 5 Department of Anatomy and Embryology, Faculty of Medicine, University of Tsukuba, Tsukuba, Ibaraki 305-8575, Japan

## Abstract

cAMP responsive element binding protein 3-like 3 (CREB3L3), a transcription factor expressed in the liver and small intestine, governs fasting-response energy homeostasis. Tissue-specific CREB3L3 knockout mice have not been generated till date. To our knowledge, this is the first study using the one-step CRISPR/Cas9 system to generate CREB3L3 floxed mice and subsequently obtain liver- and small intestine-specific *Creb3l3* knockout (LKO and IKO, respectively) mice. While LKO mice as well as global KO mice developed hypertriglyceridemia, LKO mice exhibited hypercholesterolemia in contrast to hypocholesterolemia in global KO mice. LKO mice demonstrated up-regulation of hepatic *Srebf2* and its corresponding target genes. No phenotypic differences were observed between IKO and floxed mice. Severe liver injury was observed in LKO mice fed a methionine-choline deficient diet, a model for non-alcoholic steatohepatitis. These results provide new evidence regarding the hepatic CREB3L3 role in plasma triglyceride metabolism and hepatic and intestinal CREB3L3 contributions to cholesterol metabolism.

CREB3L3 is a membrane-bound transcription factor belonging to the CREB/ATF family. *Creb3l3* is expressed in the liver and intestine^1^. Translated CREB3L3 protein localizes to the endoplasmic reticulum (ER) before transfer to the Golgi apparatus, where the transcriptionally active N-terminal region is cleaved prior to translocation to the nucleus^2^. *Creb3l3* mRNA has consistently been shown to be highly regulated by fasting and re-feeding, with nuclear levels of the active form of CREB3L3 seen to increase in times of starvation^3^. CREB3L3 and peroxisome proliferator activated receptor alpha (PPARα) synergistically activate hepatic fibroblast growth factor 21 (*Fgf21)* expression and exert effects on energy metabolism through the modulation of plasma FGF21 levels^4^,^5^. Synthesized FGF21 proteins are secreted into the circulation and have been shown to exert effects on numerous peripheral tissues including the brain, white adipose tissue (WAT), brown adipose tissue (BAT), and skeletal muscle. FGF21 activates lipolysis in WAT and thermogenesis in BAT^6^. Further, these effects alleviate the symptoms of diabetes and hyperlipidemia via reductions in plasma glucose, insulin, triglyceride (TG), and cholesterol levels. CREB3L3 reduces plasma TG levels by increasing hepatic gene expression of apolipoproteins such as apolipoprotein A-IV (*Apoa4*), *Apoa5*, and *Apoc2*^1^. These apolipoproteins activate plasma lipoprotein lipase (LPL) activity, resulting in reduced plasma TG levels.

Genetically modified mouse models represent valuable tools for the studying development and diseases. Traditional gene targeting in embryonic stem (ES) cells, although suitable for generating sophisticated genetic modifications in endogenous genes, remains complex and time-consuming. However, the production of genetically modified mice and rats has been greatly accelerated by recently developed approaches using direct injection of DNA or mRNA encoding site-specific nucleases into one-cell-stage embryos, thereby generating DNA double-strand breaks (DSBs) at specified sequences leading to targeted mutations. Co-injection of single-stranded or double-stranded DNA templates homologous to sequences flanking DSB can produce mutant alleles with precise point mutations or DNA inserts. Engineered endonucleases, including zinc-finger nucleases (ZNFs), transcription activator-like effector nucleases (TALENs), and the clustered regularly interspaced short palindromic repeat (CRISPR)/CRISPR associated protein 9 (Cas9) system have all demonstrated utility in the rapid generation of genetically modified animals. The CRISPR/Cas9 system is an RNA-mediated adaptive immune system found in bacteria and archaea that protects against the invasion of viruses and plasmids^7^. On the basis of locus organization and signature Cas gene composition, three major types of CRISPR systems (type I–III) have been identified^8^. The modified type II CRISPR/Cas9 system, derived from *Streptococcus pyogenes*, is widely used for gene editing^8^,^9^. The type II bacterial CRISPR/Cas9 system has been demonstrated as an efficient gene-targeting technology that facilitates multiplexed gene targeting. Because the binding of Cas9 is guided by simple base-pair complementarities between engineered single-guide RNA (sgRNA) and target genomic DNA sequences, it is possible to direct Cas9 to any genomic locus by providing engineered sgRNA. Previous reports have demonstrated that the generation of floxed mice in one step by the insertion of two loxP sites into the same allele of genes using the CRISPR/Cas9 system^10^,^11^,^12^. However, the generation of tissue-specific knockout (KO) mice by crossing floxed mice developed using the methods outlined above with tissue-specific Cre Tg mice has yet to reported.

Until now, CREB3L3 loss of function studies have relied on *Creb3l3*^−/−^ mice. However, the contribution of intestinal CREB3L3 to lipid metabolism has yet to be specifically examined. Accordingly, conditional *Creb3l3* knockout mice are required to evaluate the tissue-specific functions of CREB3L3. In the present study, we generated *Creb3l3* floxed mice using the one-step CRISPR/Cas9 system. For the first time, both liver-specific *Creb3l3* knockout (LKO) and intestine-specific *Creb3l3* knockout (IKO) mice were successfully generated by crossing *Creb3l3* floxed mice with albumin- and villin-promoter Cre Tg mice, respectively. Phenotypic characteristics were then compared between tissue-specific CREB3L3 KO mice.

## Results

### Generation of *Creb3l3* floxed mice using a one-step CRISPR/Cas9 system

**Figure 1:**
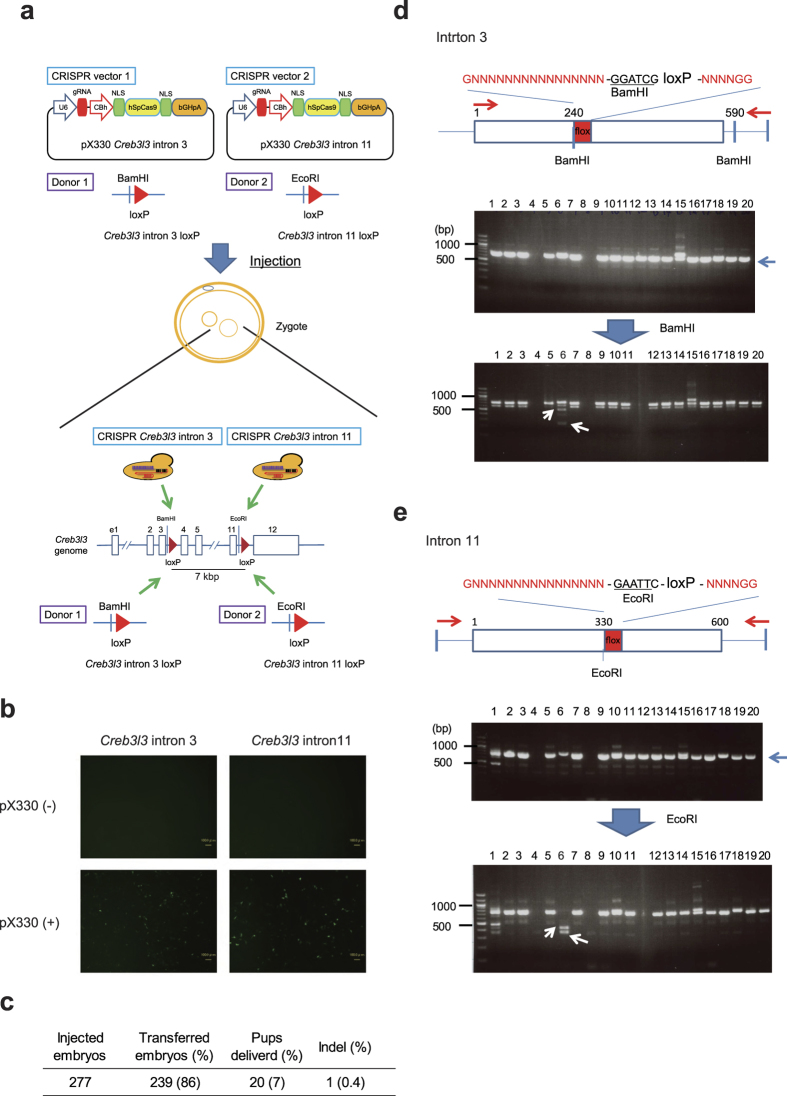
Strategy used for one step CRISPR/Cas9 generation of *Creb3l3* floxed mice. (**a**) Method used to generate *Creb3l3* floxed mice using the one-step CRISPR/Cas9 system. (**b**) *Creb3l3* CRISPR activity was evaluated using the EGxxFP system. Robust EGFP signals were observed following the co-transfection of HEK293T cells with the pCX-EGxxFP*-Creb3l3* intron vector and either the pX330 empty vector or px330*-Creb3l3* intron vector. Scale bars represent 100 μm. (**c**) Generation of mutant mice via two vectors and two DNA fragments injection. (**d,e**) Genotyping to confirm insertion of loxP sites into *Creb3l3* introns 3 (**d**) and 11 (**e**). Mouse tail genomic DNA was isolated and subjected to genotyping PCR. PCR products were cut using BamHI or EcoRI restriction enzymes.

CREB3L3 is a key regulator of glucose and lipid metabolism. Although *Creb3l3*^−/−^ mice have previously been generated^13^, the generation of conditional *Creb3l3* knockout mice has yet to be reported. Since CREB3L3 is specifically expressed in the liver and intestine, it is important to elucidate the differential functions of CREB3L3 in these organs. Accordingly, we aimed to generate *Creb3l3* floxed (floxed) mice using the CRISPR/Cas9 system. Our plan was to insert flox site in intron 3 and 11 of the *Creb3l3* gene, respectively, and then delete the about 7kbp between both sites by the Cre-loxP system. A px330 vector was used to express gRNA for the targeted region of the gene of interest (*Creb3l3* in the present study) and Cas9 protein in mouse zygotes^14^. We identified appropriate CRISPR target sites in introns 3 and 11 of the *Creb3l3* gene. Double-stranded DNAs (dsDNAs) of 20 bp were derived from regions of introns 3 and 11 of the *Creb3l3* gene and inserted into the px330 vector, with the resultant plasmids designated as px330-*Creb3l3* intron 3 and px330-*Creb3l3* intron 11, respectively. We designed corresponding loxP site oligos with 250-bp sequences homologous to each side surrounding each sgRNA-mediated DSB. To facilitate the detection of the intended insertions, DNA fragments targeting intron 3 were engineered to contain a BamHI restriction site and other oligos targeting intron 11 to contain an EcoRI site in addition to the loxP sequences, respectively (Fig. 1a and Supplementary Fig. 1).

The EGxxFP system^15^ involves the formation of a fragment of pCX-EGxxFP-*Creb3l3* cleaved from the px330-*Creb3l3* vector in order to produce full-length functional EGFP by HDR. The cleavage activity of the px330-*Creb3l3* intron 3 and px330-*Creb3l3* intron 11 vectors were confirmed using the EGxxFP system. Fragments of intron 3 and intron 11 of the *Creb3l3* gene were inserted into the multi-cloning site of pCX-EgxxFP, with the resultant vectors designated pCX-EGxxFP-*Creb3l3* intron 3 and pCX-EGxxFP-*Creb3l3* intron 11, respectively. To confirm the above mechanism, the target sequence of *Creb3l3* intron 3 or 11 for pCX-EGxxFP-*Creb3l3* and px330-*Creb3l3* vectors were co-transfected into HEK293T cells, respectively, and EGFP signals were observed in co-transfected cells. EGFP signals were clearly detected following co-transfection with both pX330 *Creb3l3* and pCX-EGxxFP vectors (Fig. 1b). We further confirmed the ability of px330-*Creb3l3* vectors to cleave both intron 3 and intron 11 of the *Creb3l3* genome, respectively.

Next, the px330-*Creb3l3* intron 3 and px330-*Creb3l3* intron 11 vectors, and two dsDNA fragments intron 3 and intron 11 containing a loxP sequence, were injected into mouse 277 zygotes and 239 embryos were transferred into the oviducts of pseudopregnant recipient ICR mice with the aim of generating 20 pups (Fig. 1c). Genomic DNA from pups born after microinjection were amplified by PCR and subjected to digestion with BamHI and EcoRI separately. Genomic DNA from pups 4 and 8 (lanes 4 and 8 in Fig. 1d,e) were not detected by genotyping PCR for intron 3 and intron 11. This result demonstrates an important issue with the one step CRISPR/Cas9 system. Introns 3 and 11 of genomic *Creb3l3* were predicted to be completely deleted in these mice. Fortunately, we identified one mouse (No. 6) which carried loxP sites at both the intron 3 and intron 11 sites of the same allele (Fig. 1d,e). It was confirmed that the loxP site within intron 3 was heterozygously inserted; however, the intron 11 site was homozygously inserted in this mouse (Fig. 1c–e).

### Generation of liver- and intestine-specific *Creb3l3* knockout mice

**Figure 2:**
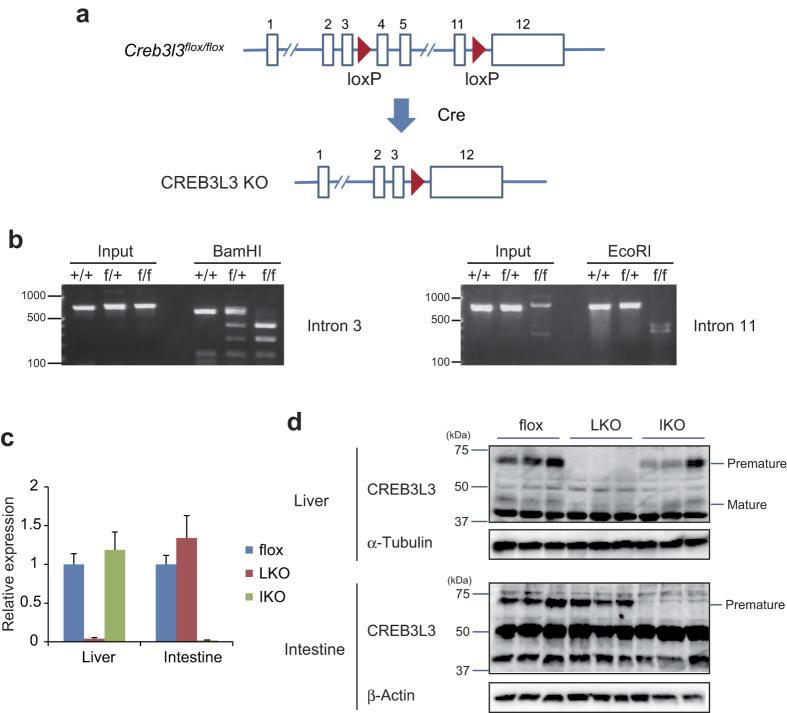
Generation of tissue-specific *Creb3l3* knockout mice. (**a**) Method used to generate tissue-specific CREB3L3 KO mice. (**b**) Genotyping of *Creb3l3*^+/+^ (+/+), *Creb3k3*^* f lox*/+^ (f/+), and *Creb3l3*^*flox/flox*^ (f/f) to confirm insertion of loxP sites into both Creb3l3 introns 3 and 11. (**c**) qPCR to measure hepatic and intestinal *Creb3l3* expression in floxed, CREB3L3 LKO, and IKO mice in fasted condition (n = 5–6 per group). (**d**) Immunoblot analysis for CREB3L3 protein in hepatic and intestinal tissues from floxed, LKO, and IKO mice in fasted condition.

As *Creb3l3* is predominantly expressed in the liver and small intestine^2^, we aimed to specifically delete *Creb3l3* in these tissues. The lox sites were homozygously inserted in both sites of genomic *Creb3l3* in the floxed mice, which were confirmed by sequencing (Supplementary Fig. 2). To analysis off-target cleavages, we searched candidates that matches 12 bases at the 3′ end and the NGG PAM sequence^15^ with CRISPR direct (https://crispr.dbcls.jp/). Here we examined 6 potential off-target sites, resulting in no mutations (Supplementary Fig. 3). To determine whether the px330-*Creb3l3* intron 3 and px330-*Creb3l3* intron 11 vectors were integrated into the chromosomes, we performed PCR assay with mouse genomic DNA and a primer pair for Cas9 detection. Cas9 PCR products were not observed in the floxed mice (Supplementary Fig. 4). Generated floxed mice were then crossed with mice expressing tissue-specific Cre Tg mice (Fig. 2a,b). We used the albumin-promoter Cre Tg mice for the generation of LKO mice and the villin-promoter Cre Tg mice for IKO mice. To confirm the generation of LKO and IKO mice, we assessed gene and protein expression of CREB3L3 in the liver and intestine using qPCR and western blotting. CREB3L3 mRNA and protein were specifically deleted in the liver of LKO mice and intestine of IKO mice, respectively (Fig. 2c,d). Levels of CREB3L3 mRNA and protein in the intestine of LKO mice and liver of IKO mice were similar to levels measured in CREB3L3 floxed mice. As a result, we obtained tissue-specific CREB3L3 knockout mice using a one-step injection method in markedly shorter time than reported for previous techniques. To our knowledge, the present study represents the first report of the use of the one-step CRISPR/Cas9 system in generating tissue-specific knockout mice. Although previous reports have demonstrated the generation of floxed mice, none have reported the application of this approach to the generation of knockout mice^10^.

### Liver-specific *Creb3l3* knockout mice and not intestine-specific knockout mice demonstrate hyperlipidemia

**Figure 3:**
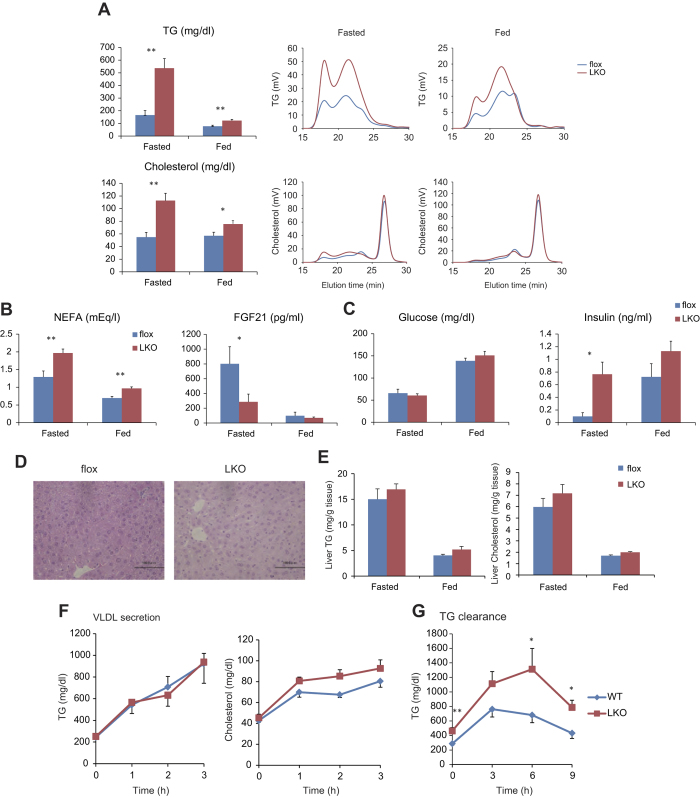
Plasma parameters of CREB3L3 LKO mice. (**A**) Plasma TG and cholesterol, and HPLC analysis of plasma TG and cholesterol levels (**B**) NEFA, and plasma FGF21 levels, and (**C**) glucose, and insulin levels in 8-week-old male floxed and CREB3L3 LKO mice in fasted and fed conditions. (**D**) Histological analyses of liver sections stained with HE from 8-week-old male floxed and CREB3L3 LKO mice in fasted condition. (**E**) Liver TG and cholesterol levels in floxed and CREB3L3 LKO mice in fasted and fed conditions. (**F**) VLDL secretion analysis. Eight week-old male floxed and LKO mice were fasted for 18 h prior to the administration of Triton WR-1339 with blood collected at the indicated times. Plasma TG and cholesterol levels were evaluated. (**G**) TG clearance assays. Mice were fasted for 24 h prior to oral administration of olive oil with blood collected at the indicated time for plasma TG level measurements (n = 5–10 per group). *p < 0.05 and **p < 0.01.

No significant differences in body weight or lean weight were observed between floxed, LKO, and IKO mice in both fasted and fed states, except an increased weight proportion attributable to fat in IKO mice in the fed state compared to floxed mice as estimated by DEXA scanning (Supplementary Table 1). Plasma TG and glucose levels have been reported to be higher, with cholesterol and FGF21 levels reported lower, in *Creb3l3*^−/−^ mice compared to WT mice^5^,^16^. Plasma TG, cholesterol, and free fatty acid levels were significantly increased in LKO mice compared to floxed mice in both fasted and fed conditions (Fig. 3A,B). High-performance liquid chromatography (HPLC) demonstrated CM, VLDL, and LDL fractions of TG were markedly increased in LKO mice in both fasted and fed states (Fig. 3A). Plasma FGF21 levels were markedly decreased in LKO mice in the fasted state, but were unchanged in the fed state (Fig. 3B). No difference in plasma glucose levels were observed between LKO and floxed mice (Fig. 3C). Plasma insulin levels were increased in LKO mice in both fasted and fed states, but not significantly in fed state (Fig. 3C). No changes in plasma parameters were observed in IKO mice, except for a decrease in plasma glucose in the fed condition compared to floxed mice (Supplementary Table 2). Histological analyses of liver sections following H&E staining, demonstrated no apparent morphological differences between LKO and floxed mice (Fig. 3D). No difference in hepatic lipid content, including TG and cholesterol, were observed between floxed and LKO mice in both fasted and fed states, with no difference in liver weights observed (Fig. 3E and Supplementary Table 1). LKO mice have higher plasma cholesterol levels and *Creb3l3*^−/−^ mice have lower^1^, while IKO mice have no difference compared with control mice, indicating that CREB3L3 in enterohepatic circulation plays a pivotal role in cholesterol metabolism.

### Liver-specific *Creb3l3* knockout mice have dysfunctional TG clearance but not VLDL secretion

Previous reports have described lower plasma LPL activity in *Creb3l3*^−/−^ mice leading to the lower TG clearance compared to WT mice, with no effect on VLDL secretion^1^. LKO mice were subjected to evaluation of VLDL secretion and TG clearance. In VLDL secretion tests, no difference in plasma TG levels was observed between LKO and floxed mice, indicating normal VLDL secretion in LKO mice (Fig. 3F). There was a trend toward increased plasma cholesterol levels in LKO mice compared to floxed mice from 1 h after injection (Fig. 3F). In TG clearance testing, plasma TG levels were significantly higher in LKO mice compared to floxed mice (Fig. 3G). These results indicate that the dysregulation of TG clearance in LKO mice can be explained by hepatic *Creb3l3* deficiency.

### Hepatic deletion of *Creb3l3* dysregulates the expression of genes related to lipid metabolism

**Figure 4:**
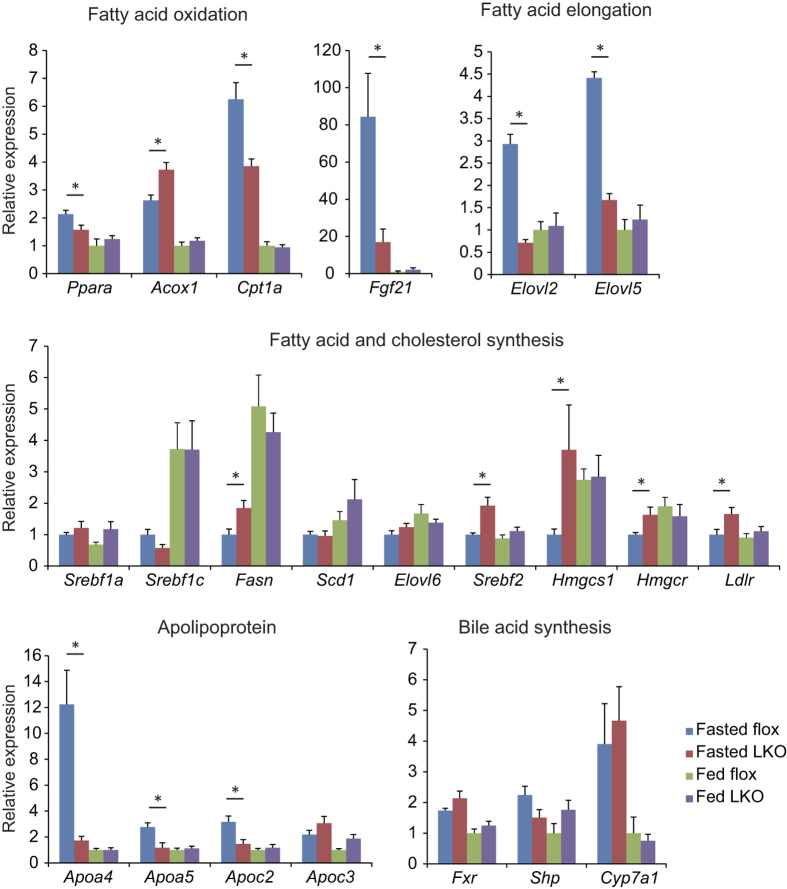
Hepatic gene expression profiles in CREB3L3 LKO mice. Hepatic gene expression profiles in 8-week-old male floxed and LKO mice in fasted and fed conditions (n = 4–7 per group). *p < 0.05 and **p < 0.01.

Next we evaluated the expression of genes related to lipid metabolism including TG, cholesterol, and fatty acids in the livers of LKO mice in fasted and fed states (Fig. 4). No significant changes in gene expression levels were observed in the fed state. CREB3L3 is predominantly active in the fasted state, rather than the fed state. Thus, this result was expected. In the fasted state, expression levels of *Ppara*, another key regulator of fatty acid oxidation, were reduced in LKO mice. Consistently, hepatic expression levels of the PPARα target gene, carnitine palmitoyltransferase 1a (*Cpt1a*), were also reduced. The expression of another target gene, acyl-Coenzyme A oxidase 1, palmitoyl (*Acox1*), were significantly increased. No differences in the expression levels of regulators of fatty acid synthesis, such as sterol regulatory element binding transcription factor 1a (*Srebf1a*) and *Srebf1c*, and their corresponding target genes, stearoyl-Coenzyme A desaturase 1 (*Scd1*), and ELOVL family member 6, elongation of long chain fatty acids (yeast) (*Elovl6*), were observed between floxed and LKO mice in either nutritional state, but fatty acid synthase (*Fasn*) was significantly increased. On the other hand, the cholesterol synthesis regulator, *Srebbf2*, was significantly increased in the fasted state, leading to an increase in its corresponding target genes including 3-hydroxy-3-methylglutaryl-Coenzyme A synthase 1 (*Hmgcs1*), 3-hydroxy-3-methylglutaryl-Coenzyme A reductase (*Hmgcr*), and low density lipoprotein receptor (*Ldlr*). Consistent with a previous report in *Creb3l3*^−/−^ mice^16^, fatty acid elongases, such as *Elovl2* and *Elovl5*, were significantly decreased in LKO mice in the fasted state indicating a changed composition of fatty acids in the livers of LKO mice. Of the examined lipoprotein lipase (LPL) activity regulators, expression levels of activators such as *Apoa4*, *Apoa5*, and *Apoc2* were significantly reduced, while expression levels of a suppressor, *Apoc3*, were conversely increased, not significant, in livers of LKO mice compared to floxed mice in the fasted state. These results provide evidence of the dysregulation of TG clearance in LKO mice, consistent with previously reported phenotypes of *Creb3l3*^−/−^ mice. No significant differences in expression levels of bile acid metabolic genes, nuclear receptor subfamily 1, group H, member 4 (*Fxr* encoded by *Nr1h4*), small heterodimer partner (*Shp*), or cytochrome P450, family 7, subfamily a, polypeptide 1 (*Cyp7a1*) were observed between LKO and floxed mice. In consistent with previous reports about *Creb3l3*^−/−^ mice^1^,^4^,^5^,^14^, changes in the hepatic expression of genes related to fatty acid oxidation, fatty acid elongation, and apolipoprotein in LKO mice were same as *Creb3l3*^−/−^ mice. LKO mice especially exhibited the increased hepatic expression of genes related to cholesterol synthesis. Given these changes in gene expression, we suggest the increased hepatic cholesterol synthesis and reductions in plasma LPL activity observed in LKO mice contribute to hyperlipidemia.

### Deficiency of *Creb3l3* in the liver accelerates non-alcoholic steatohepatitis

**Figure 5:**
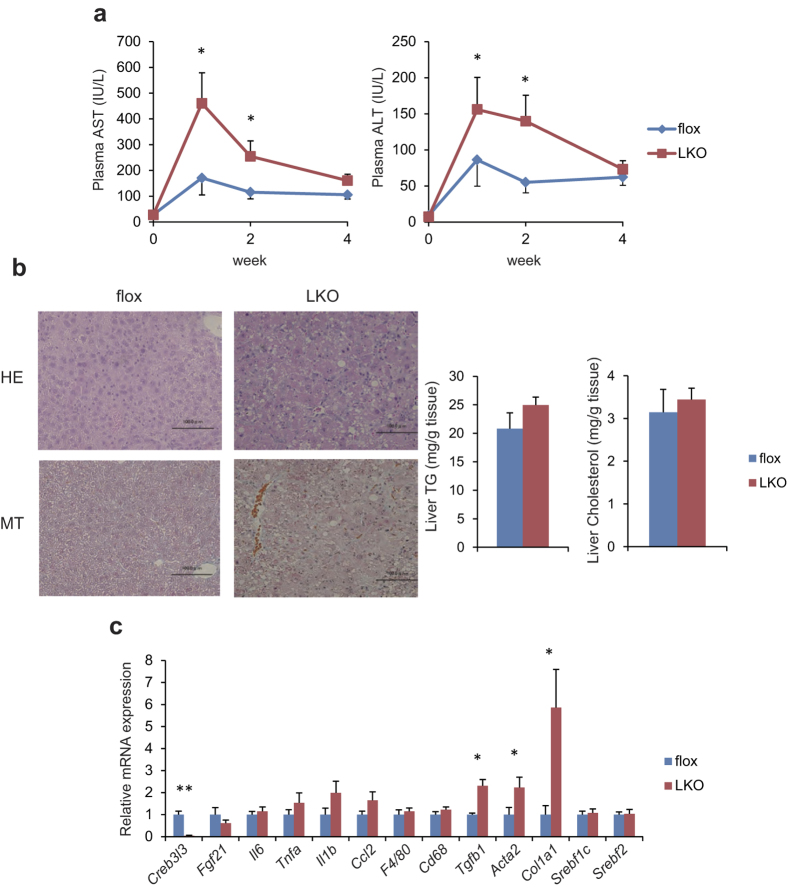
Hepatic *Creb3l3* deficiency aggravates MCD diet-induced liver injury and fibrosis. Eight-week-old male mice were fed the MCD diet for 4 weeks. (**a**) Plasma AST and ALT levels during feeding on MCD diet. (**b**) Hepatic histological analysis (HE staining, and MT staining) and hepatic lipid contents in floxed and LKO mice fed the MCD diet for 4 weeks. (**c**) Hepatic gene expression profiles of floxed and LKO mice fed the MCD diet for 4 weeks (n = 5–10 per group). *p < 0.05 and **p < 0.01.

To determine the effects of hepatic CREB3L3 on NASH pathology, LKO mice were fed the methionine-choline deficient (MCD) diet for 4 weeks leading to the induction of non-alcoholic fatty liver disease (NAFLD). Plasma alanine aminotransferase (ALT) and aspartate aminotransferase (AST) levels were acutely and significantly increased in LKO mice fed an MCD diet, indicating severe and acute liver injury (Fig. 5a). Histological analysis of sections stained with H&E demonstrated disrupted hepatic architecture in LKO mice, with Masson’s trichrome (MT) staining demonstrating greater induction of fibrosis in LKO mice compared to floxed mice (Fig. 5b). Surprisingly, no differences in hepatic TG and cholesterol contents were observed between floxed and LKO mice (Fig. 5c). Consistent with the results of the MT staining analysis, hepatic gene expression studies demonstrated significantly increased hepatic expression of fibrosis-related genes such as transforming growth factor, beta 1 (*Tgfb1*), actin, alpha 2, smooth muscle, aorta (*Acta2*), and collagen, type I, alpha 1 (*Col1a1*) in LKO mice (Fig. 5c). There was a trend toward decreased hepatic expression of the CREB3L3 target gene, In FGF21 KO mice fed the MCD diet, non-alcoholic steatohepatitis (NASH) phenotypes were induced^17^. *Fgf21* was decreased in LKO mice, thereby in part contributing to the pathology of NASH. There was a trend toward increased expression levels of inflammatory mediators including *Il6*, *Il1b*, and *Ccl2*; however, these differences were not statistically significant. Interestingly, no significant differences were observed in the expression levels of fatty acid and cholesterol synthesis regulator genes, such as *Srebf1c* and *Srebf2*, between LKO and floxed mice (Fig. 5c). These results indicate hepatic *Creb3l3* deficiency acutely exacerbates MCD diet-induced liver injury and fibrosis, without abnormal hepatic lipid accumulation.

## Discussion

There are three previous studies reporting the generation of conditional mutant mice by introduction of two flox sites using the CRISPR/Cas9 system^10^,^11^,^12^. However, no reported studies have used the one-step CRISPR/Cas9 system to generate floxed mice and subsequently tissue-specific knockout mice. The present study is the first to report the generation of tissue-specific knockout mice and to analyze the function of the gene of interest in mice. We generated *Creb3l3* floxed mice by direct microinjection of 2 vectors and 2 DNA fragments into C57BL/6J zygotes using the CRISPR/Cas9 system. *Creb3l3* is predominantly expressed in the liver and small intestine. We intended to generate liver-specific and intestine-specific *Creb3l3* knockout mice by crossing *Creb3l3* floxed mice with tissue-specific promoter-driven Cre Tg mice, respectively. In contrast to *Creb3l3*^−/−^ mice reported to have high plasma TG levels only^1^, LKO mice demonstrated higher plasma TG and cholesterol levels but not hepatic steatosis in the fasting condition. These changes were considered to be attributable to intestinal expression of *Creb3l3* in addition to hepatic expression. Collectively, we propose that CREB3L3 functions as a mediator of lipid metabolism via regulation of the enterohepatic circulation.

Previous reports have demonstrated the generation of floxed mice using the one-step CRISPR/Cas9 system; however, no studies have demonstrated the generation of tissue specific knockout mice^10^. The major limitation of the one step system is that in situations where 2 sites at the same allele are ligated, the genomic portion between 2 sites could be deleted leading to failed insertion of the loxP sites. Furthermore, the frequency of the insertion of 2 loxP sites into the same allele may be extremely low. We obtained only one line of *Creb3l3* floxed mice with 2 loxP sites within the same allele.

We generated both LKO and IKO mice through breeding with mice generated using the one step CRISPR/Cas9 system. IKO mice had no apparent differences in metabolic parameters compared to floxed mice, while LKO mice demonstrated hyperlipidemia. These findings indicate hepatic CREB3L3 contributes to cholesterol and TG homeostasis. Consistent with previous reports, hepatic expression of LPL activator genes was reduced and suppressor genes was increased in LKO mice, underlying the observed increases in plasma TG levels in LKO mice. Hepatic *de novo* cholesterol synthesis was apparently activated leading to increased plasma cholesterol levels in LKO mice. Accordingly, hepatic expression levels of genes related to cholesterol synthesis were higher in LKO mice compared to floxed mice. Despite the higher plasma cholesterol levels in LKO mice, no differences in hepatic cholesterol content were observed between floxed and LKO mice. Expression levels of *Srebf2* are known to be regulated by a feedback loop between gene expression and cholesterol levels. Therefore, increased expression of *Srebf2* and its corresponding target genes led to up-regulation of *de novo* cholesterol synthesis in LKO mice, resulting in increased plasma cholesterol levels. SREBP-2, a regulator of the expression of genes involved in cholesterol synthesis, is similar to CREB3L3 as both molecules have a transmembrane domain cleaved by site-1 and site-2 proteases. We hypothesized that CREB3L3 and SREBP-2 compete as targets of these cleavage enzymes, the detailed mechanism of which is described in a manuscript currently under submission (Nakagawa Y, paper in preparation). In addition, FGF21 suppresses *Srebf2* expression in the liver of *Apoe*^−/−^ mice^18^. Plasma FGF21 levels were lower in LKO mice, but not IKO mice than floxed mice. The results of the present study indicate liver-specific deletion of CREB3L3 leads to activation of *Srebf2* expression and the SREBP cleavage process resulting in the stimulation of SREBP-2 protein activity. These changes were shown in only LKO mice, not *Creb3l3*^−/−^ mice, thereby supporting that the function of CREB3L3 in enterohepatic circulation contributes to the regulation of hepatic SREBP2 activation.

We hypothesized that lipid metabolism dysregulation in LKO mice influences the pathogenesis of NAFLD in response to the MCD diet. Surprisingly, LKO mice demonstrated increased liver injury and fibrosis in the absence of the excess hepatic lipid accumulation compared to floxed mice, indicating liver-specific deletion of *Creb3l3* accelerates the development of non-alcoholic steatohepatitis. MCD diet-fed *Fgf21*^−/−^ mice also exhibit NASH phenotypes^17^, thereby supporting that a decrease of *Fgf21* in LKO contributes to same phenotypes. In addition, we are aware of data demonstrating increased inflammation and infiltration of macrophages in *Creb3l3*^−/−^ mice fed the MCD diet along with liver injury and fibrosis (Nakagawa Y, unpublished data). NAFLD patients, and in particular patients with NASH, are more likely to exhibit increased intestinal permeability compared with healthy controls^19^. Likewise, MCD-fed mice develop intestinal permeability changes after an initial phase of liver injury^19^. NASH is associated with increased intestinal permeability suggesting the early phase of liver injury and inflammation contributes to injury to the intestinal barrier, thereby indicating the contribution of gut–liver crosstalk in NASH pathogenesis^20^. Taken together, these results indicate enhanced disease progression in *Creb3l3*^−/−^ mice is due to aberrant inflammation in mice with intestine-specific deletion of CREB3L3 in addition to mice with liver-specific deletion of CREB3L3. According, CREB3L3 may contribute to the pathogenesis of metabolic syndrome through its role in regulating enterohepatic circulation. However, further studies are required to verify the intestinal function of CREB3L3 in models of metabolic disease such as those fed the MCD diet.

The present study demonstrated the utility of the one-step CRISPR/Cas9 system as a simple and less time-consuming method for generating genetically modified mouse models. The findings of the present study further demonstrate a crucial role for CREB3L3 in the pathogenesis of NAFLD through its function in regulating enterohepatic circulation. Future studies using intestine-specific *Creb3l3* knockout mice may demonstrate CREB3L3 as a therapeutic target for improving hyperlipidemia.

## Experimental procedures

### Animals

This project was approved and performed in accordance with the guidelines of the Animal Care Committee of the University of Tsukuba. *Creb3l3*^*tm1.1Sad*^/J (*Creb3l3*^−/−^)^13^, B6.Cg-Tg (Alb-cre) 21Mgn/J (Albumin Cre Tg)^21^, and B6.Cg-Tg (Vil1-cre) 1000Gum/J (Villin Cre Tg) mice were purchased from Jackson Laboratory. For the NAFLD study, eight-week-old male mice were fed the MCD diet (Oriental Yeast, Tokyo, Japan) for 4 weeks. Fat and lean mass analyses were performed using dual-energy X-ray absorptiometry (DEXA) with a PIXImus mouse densitometer (GE Medical Systems Lunar). All animal husbandry procedures and animal experiments were consistent with the University of Tsukuba’s Regulation of Animal Experiment and were approved by the Animal Experiment Committee, University of Tsukuba.

### Vector construction

The px330 vector (Addgene plasmid 42230) was a gift from Dr. Feng Zhang^14^. *Creb3l3* intron 3 CRISPR F (5′-caccGTGTATGTGTGCCATAGCACAGG-3′) and *Creb3l3* intron 3 CRISPR R (5′-aaacCCTGTGCTATGGCACACATACAC-3′), *Creb3l3* intron 11 CRISPR F (5′-caccGATACCAGGGGTGATGGTGCAGG-3′) and *Creb3l3* intron 11 CRISPR R (5′-aaacCCTGCACCATCACCCCTGGTATC-3′) oligo DNAs were annealed using standard methods. Annealed DNA was purified by ethanol precipitation. Short double-stranded DNA fragments were inserted into the BbsI restriction site of the px330 vector. Constructed plasmids were designated px330-*Creb3l3* intron 3 and px330-*Creb3l3* intron 11, respectively. For the EGxxFP system^15^, regions of intron 3 and intron 11 of the *Creb3l3* gene were amplified using standard PCR methods using the following primers: *Creb3l3* intron 3-EGxxFP-F: 5′-GATATCACACTGAGTTCACATAG-3′, *Creb3l3* intron 3-EGxxFP-R: 5′-GATATCAAACAGGAGGTCAAGGAC -3′, *Creb3l3* intron 11-EGxxFP-F: 5′-GATATCTAAAAAAATTTAGATAAAAGAAAAAG-3′, and *Creb3l3* intron 11-EGxxFP-R: 5′-GATATCTGGGACTTAAACTCCAGTTCTCGGC-3′. PCR products were inserted into the pCX-EGxxFP plasmid. Constructed plasmids were designated pCX-EGxxFP-*Creb3l3* intron 3 and pCX-EGxxFP-*Creb3l3* intron 11, respectively.

### Transfection

The px330 and pCX-EGxxFP vectors were co-transfected into HEK293T cells using X-treme 9 (Roche).

### Microinjection

Female C57BL/6J mice were injected with pregnant mare serum gonadotropin (PMSG) and human chorionic gonadotropin (hCG) at 48-h intervals and mated with male C57BL/6J mice. Fertilized one-cell embryos were collected from oviducts. Then, 5 ng/μl of px330 vector (circular) and 10 ng/μl dsDNA donors were injected into the pronuclei of one-cell-stage embryos according to standard protocols (Gordon and Ruddle 1981). Injected one-cell embryos were then transferred into pseudopregnant ICR mice.

### Genotyping for *Creb3l3* conditional knockout mice

Genomic DNA was obtained from tail clippings and PCR was performed using EX Taq (Takara Bio) and the following primers: Typing *Creb3l3* intron 3 F: 5′-ATCACACTGAGTTCACATAG-3′, Typing *Creb3l3* intron 3 R: 5′-TAAGCAGATGTGTGAGGGTTACAG-3′, Typing *Creb3l3* intron 11 F: 5′-AGGGGCAGGACAGTGGTCTGCTATG-3′, Typing *Creb3l3* intron 11 R: 5′-ATCTACTCTCTGAGCCATCCGCCTCAAG-3′. Amplified DNA fragments of *Creb3l3* intron 3 and intron 11 were cut using BamHI and EcoRI, respectively, and subjected to electrophoresis on an agarose gel.

### Off-target analysis

Potential off-target sites were found using CRISPR target with rules outlined previously^15^. Thirteen bases proceeding the PAM sequence with NGG were aligned with mouse genome. The genome fragments containing the off-target were sequenced.

### Detection of Cas9 DNA

Genomic DNA was obtained from tail clippings and PCR was performed using EX Taq (Takara Bio) and the following primers: Cas9 F: 5′-AGTTCATCAAGCCCATCCTG-3′, Cas9 R: 5′-GAAGTTTTCTGTTGGCGAAGC-3′. Amplified DNA fragments of Cas9 were subjected to electrophoresis on an agarose gel.

### Determination of metabolic parameters

Plasma glucose, TG, cholesterol, NEFA, AST, and ALT concentrations were measured using Wako enzymatic kits. Plasma insulin was measured using mouse insulin ELISA Kits (Sibayagi). Plasma FGF21 concentrations were measured using a mouse/rat FGF21 Quantikine ELISA assay (R&D Systems). Hepatic TG and cholesterol levels were measured using previously described protocols^22^.

### High-performance Liquid Chromatography Analysis

For lipoprotein distribution analyses, pooled plasma samples from 4 to 5 mice per group were analyzed using an upgraded high-performance liquid chromatography (HPLC) technique as previously described (Skylight Biotech)^23^.

### Histological analysis

Harvested livers were fixed, embedded in paraffin, sectioned, and stained with hematoxylin and eosin (HE) or Masson’s trichrome (MT).

### Immunoblotting

Total cell lysates from mouse livers were prepared as previously described^22^, separated by SDS-PAGE, and subjected to Western blot analysis using anti-CREB3L3^5^, α-Tubulin (05-829, Millipore), and β-Actin (sc-47778 Santa Cruz) antibodies.

### TG Production assay

Mice were fasted for 24 h prior to injection with Triton WR-1339 (0.5 mg/g body weight, Sigma-Aldrich) via the tail vein to block the clearance of nascent ApoB-containing lipoproteins. Blood samples were collected at 0, 30, 60, and 120 min after Triton injection^23^.

### Postprandial TG Response assay

Mice were fasted for 16 h prior to the oral administration of 200 μl olive oil^1^. Blood samples were collected at 0, 3, 6, and 9 h after olive oil administration.

### Analysis of gene expression

Total RNA from cells and tissues was prepared using Trizol reagent (Invitrogen). Templates for real-time PCR analysis were prepared by cDNA synthesis (Invitrogen) using total RNA. Real-time PCR was performed using the ABI Prism 7300 System (ABI) and SYBR Green Master Mix (Roche)^24^. Primer sequences are listed in Supplementary Table 3.

### Statistical analyses

Statistical significance was determined using unpaired Student’s t-tests. Differences with P < 0.05 were considered significant. Data are expressed as the mean ± SEM.

## Additional Information

**How to cite this article**: Nakagawa, Y. *et al*. Hyperlipidemia and hepatitis in liver-specific CREB3L3 knockout mice generated using a one-step CRISPR/Cas9 system. *Sci. Rep.* **6**, 27857; doi: 10.1038/srep27857 (2016).

## Supplementary Material

Supplementary Information
