# Supplementary material for: Hyperlipidemia and hepatitis in liver-specific CREB3L3 knockout mice generated using a one-step CRISPR/Cas9 system
Source: Sci Rep. 2016 Jun 13;6:27857. doi: 10.1038/srep27857 (PMC4904192; doi:10.1038/srep27857)
Supplement: Supplementary Information [file srep27857-s1.pdf]

Supplementary Section to:

Hyperlipidemia and hepatitis in liver-specific CREB3L3 knockout mice generated using a one-step CRISPR/Cas9 system

Yoshimi Nakagawa,[1,2,*] Fusaka Oikawa,[1] Seiya Mizuno,[3] Hiroshi Ohno,[1] Yuka Yagishita,[1] Aoi Satoh,[1] Yoshinori Osaki,[1] Kenta Takei,[1] Takuya Kikuchi,[1] Song-iee Han,[1] Takashi Matsuzaka,[1] Hitoshi Iwasaki,[1] Kazuto Kobayashi,[1] Shigeru Yatoh,[1] Naoya Yahagi,[1] Hiroaki Suzuki,[1] Hirohito Sone,[4] Satoru Takahashi,[2,3,5] Nobuhiro Yamada,[1] Hitoshi Shimano[1,2,*]

1Department of Internal Medicine (Endocrinology and Metabolism), Faculty of Medicine, University of Tsukuba, Tsukuba, Ibaraki 305-8575, JAPAN

2International Institute for Integrative Sleep Medicine (WPI-IIIS), University of Tsukuba, Tsukuba, Ibaraki 305-8575, JAPAN

3Laboratory Animal Resource Center, University of Tsukuba, Tsukuba, Ibaraki 305-8575, Japan

4Department of Hematology, Endocrinology and Metabolism, Niigata University Faculty of Medicine, Niigata, Niigata 951-8510, Japan

5Department of Anatomy and Embryology, Faculty of Medicine, University of Tsukuba, Tsukuba, Ibaraki 305-8575, Japan

*Correspondence: ynakagawa@md.tsukuba.ac.jp (Y.N), hshimano@md.tsukuba.ac.jp (H.Shimano)

a

GATatcacactgagttcacatagccttccagggacttcaggacagtttgatccaggcaccaatatctcctctggaattagggtttctctgtgt
EcoRV
agccctggctggcctcgaattcacagagatctgcctgcctctgcctcccgagtactgggattaaaggtgtgagccagcctctctacttgttgt
tataaacatatatgttaaaaattttaactatagtgtatgtgtgccatagGGATCCataacttcgtataatgtatgctatacgaagttatcacag
BamHI
gagccatatggatttctaaatttttggtttgttttgcttgttctttgttttgagtcttgctagtggtagaccaggtttacagactcttggttaccccagt
ccaaccctgcttacatttcaggcaGATtcacagagacccagcccgggggctgaatggaagtcacatttggcctggaaggagctggggt
gctgtgtataagaacagagtcctctctctctctctctcctggatctagatggcgtcccctgtctgtgctatggcgcccttggacagcatgga
ggtccttgacctcctgtttgatATC
EcoRV

b

GATatctaaaaaaatttagataaaagaaaaagtctcagcctcctcctgagacccacactctgatagctggaccccagcttccctgcctgtg
EcoRV
accccaccccatctccggcaggtccttctgctgtccttcgccctgatcatcctcccctccatcagccctttcaactccaacaaagttgacagc
cctggagacttcgtgccagtgcgaggtaggcagtaccagccttcggatccccagggtgagggtgaggtgccaggggcaggacagtggt
ctgctatgcagggccttggcttccattcccagcatcataGataccaggggtgatggGAATTCataacttcgtataatgtatgctatacg
EcoRI
aagttattgcaggcctgtcatacagctctgggaaggtggaggcaggggattacttcagagctatccttggctacataggacaacttgggcta
agtgagacccctgacaaaacatgtcaacacacacacacccctcaaaaccaaaagcccataccatatcaggagacttgaggcggatggct
cagagagtagatacttgccactaagcctgagaactggagtttaagtcccagatATC
EcoRV

Supplementary Figure 1 The sequences of donor *Creb3l3* intron 3 and 11 fragments containing loxP sequence. *Creb3l3* intron 3 (a) and intron 11 (b) fragments. The red character is shown loxP sequence. Underbars are shown the restrict enzyme sites.

Creb3l3 intron 3

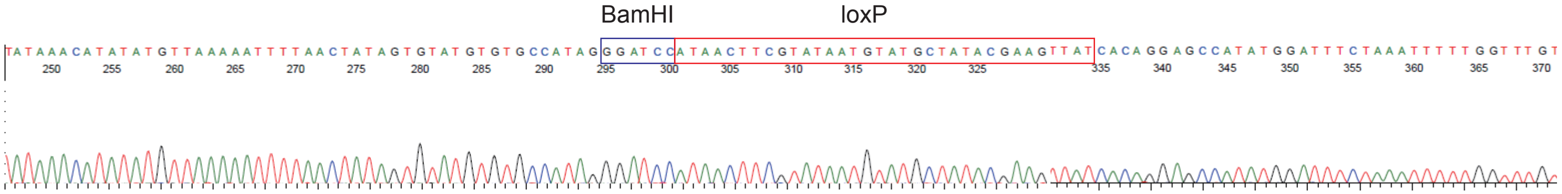

Creb3l3 intron 11

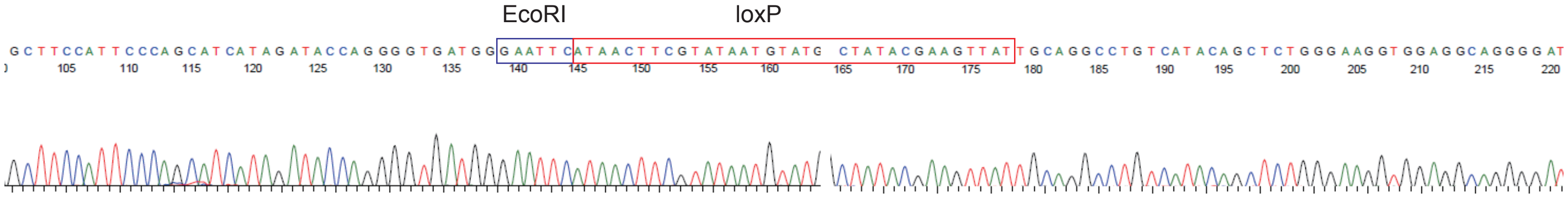

Supplementary Figure 2 The integration of loxP sites in the intron 3 and 11 of the *Creb3l3* genome. The sequences across the targeting regions confirmed correct fusion of loxP sites to the intron 3 and 11 of the *Creb3l3* genome.

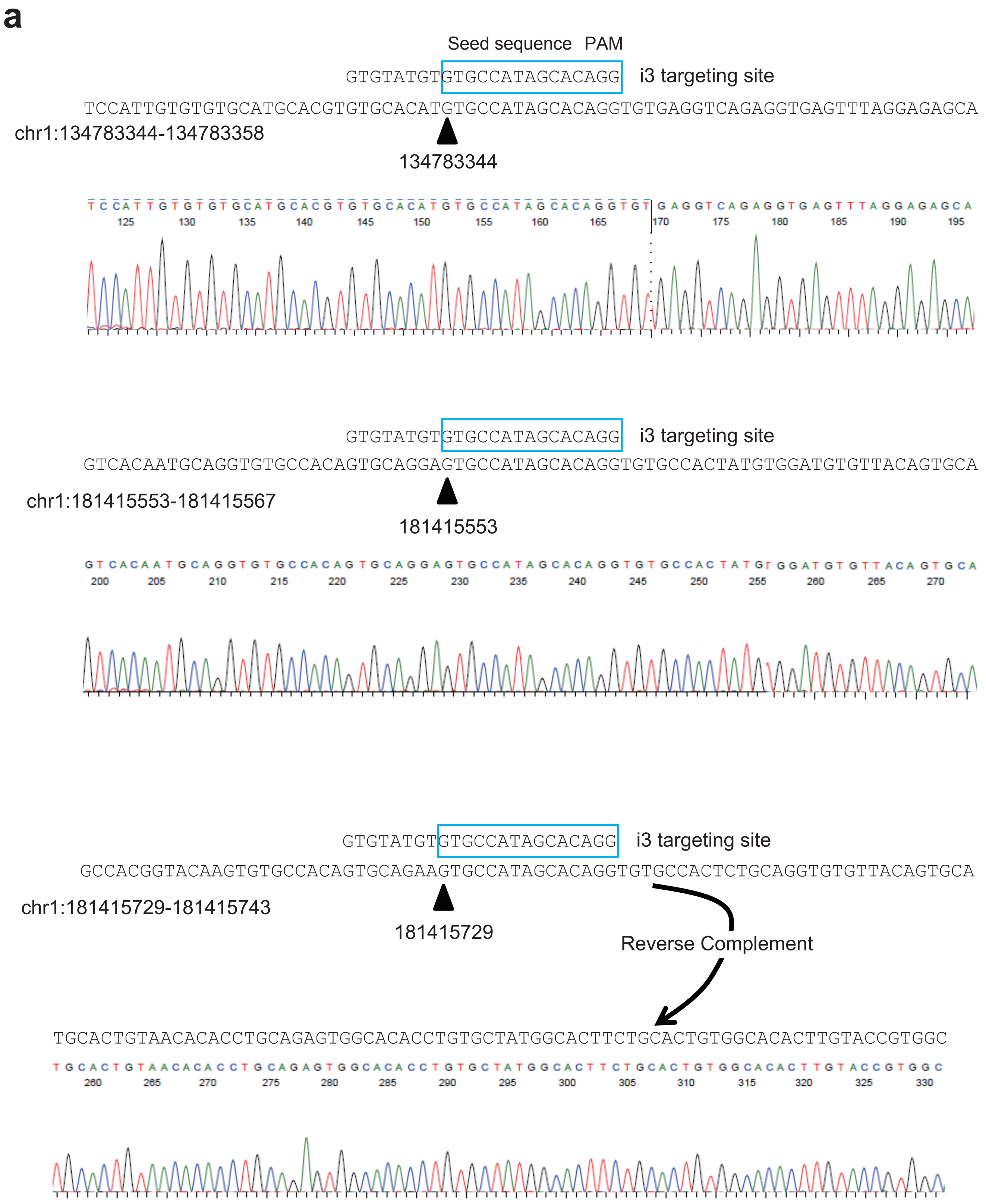
a
Seed sequence PAM
GTGTATGTGTGCCATAGCACAGG i3 targeting site
TCCATTGTGTGTGCATGCACGTGTGCACATGTGCCATAGCACAGGTGTGAGGTCAGAGGTGAGTTTAGGAGAGCA
chr1:134783344-134783358
134783344
GTGTATGTGTGCCATAGCACAGG i3 targeting site
GTCACAATGCAGGTGTGCCACAGTGCAGGAGTGCCATAGCACAGGTGTGCCACTATGTGGATGTGTTACAGTGCA
chr1:181415553-181415567
181415553
GTGTATGTGTGCCATAGCACAGG i3 targeting site
GCCACGGTACAAGTGTGCCACAGTGCAGAAGTGCCATAGCACAGGTGTGCCACTCTGCAGGTGTGTTACAGTGCA
chr1:181415729-181415743
181415729
Reverse Complement
TGCACTGTAACACACCTGCAGAGTGGCACACCTGTGCTATGGCACTTCTGCACTGTGGCACACTTGTACCGTGGC

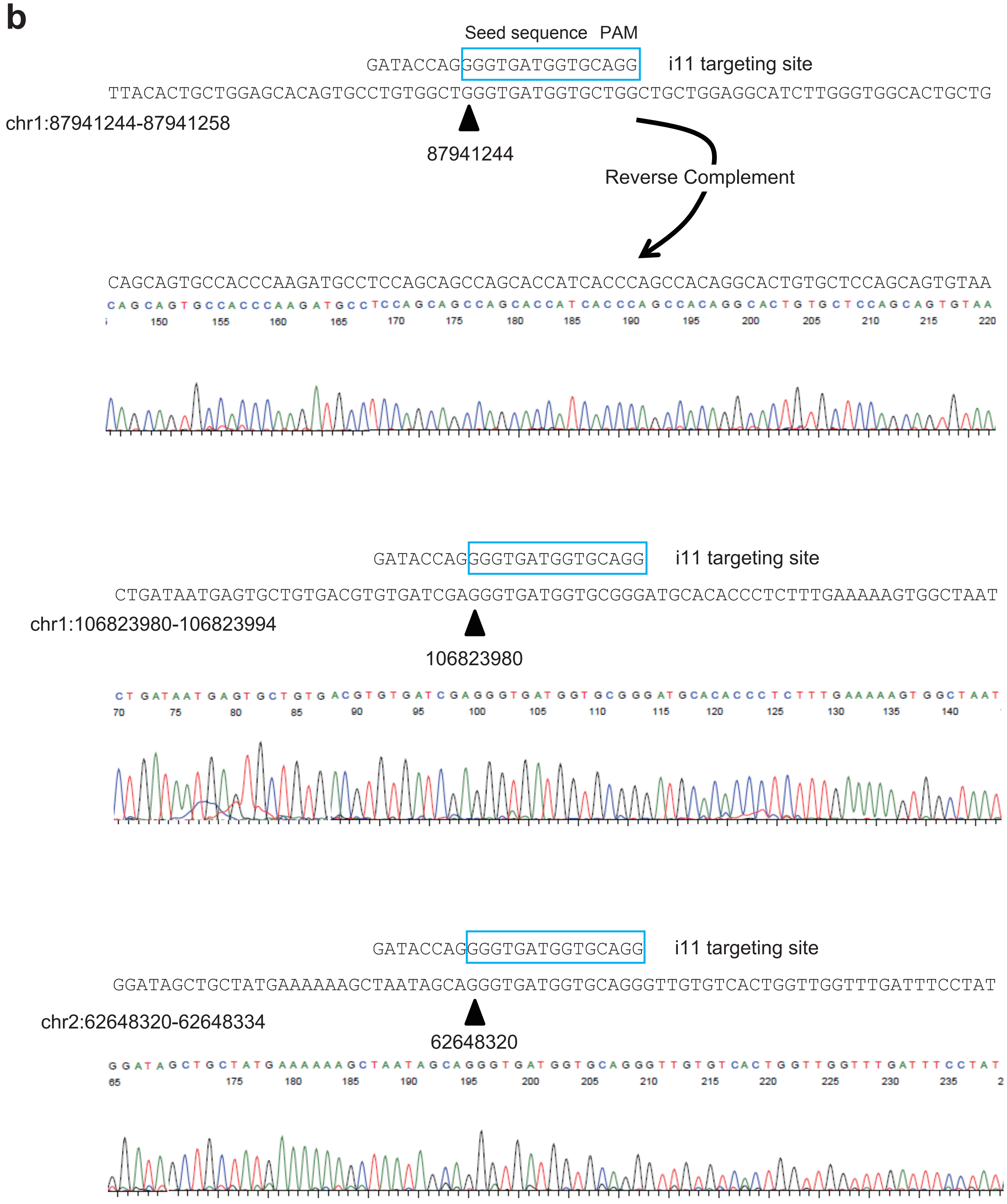

Supplementary Figure 3 Analysis of the off-target effect. The off-target sites that exactly match 12 bases at the 3' end and the NGG (blue box) were searched with free software CRISPR direct. .The correct sequences around the off-target candidates against intron 3 (a) and 11 (b) of the *Creb3l3* genome were confirmed by sequencing.

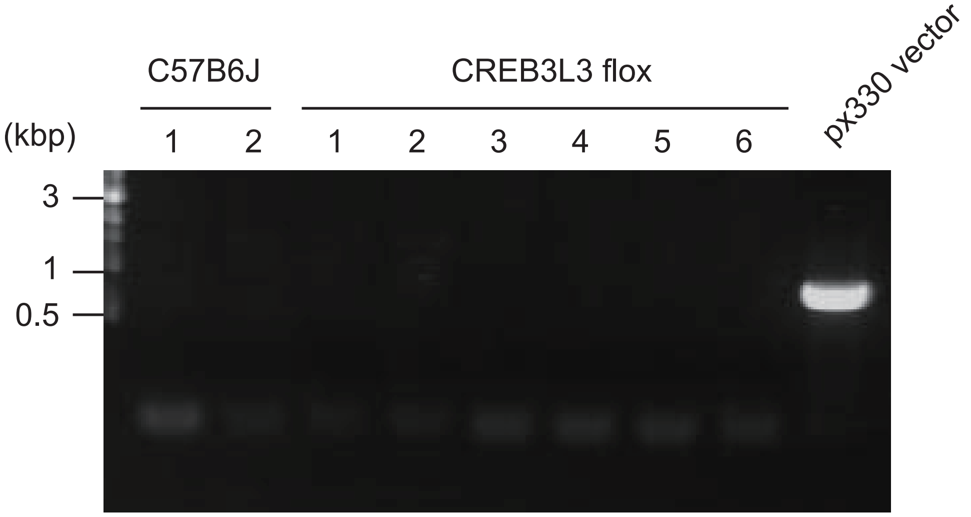

Supplementary Figure 4 The analysis of integration of pX330 vector into mouse genome.

The Cas9 cDNA were not observed in genome from CREB3L3 floxed mice by PCR.

	Fasted			Fed		
	flox	LKO	IKO	flox	LKO	IKO
BW (g)	22.83 ± 0.71	23.72 ± 0.39	21.95 ± 0.96	26.03 ± 0.92	26.69 ± 0.46	24.55 ± 1.16
Lean wight (g)	19 ± 0.69	19.62 ± 0.33	18.15 ± 0.87	21.9 ± 0.87	22.19 ± 0.43	20.2 ± 1.01
Fat weight (g)	3.83 ± 0.09	4.1 ± 0.16	3.73 ± 0.09	4.13 ± 0.12	4.49 ± 0.19	4.35 ± 0.15
Fat weight (%)	16.81 ± 0.56	17.29 ± 0.55	17.05 ± 0.36	15.94 ± 0.55	16.84 ± 0.66	17.675 ± 0.23*

Supplementary Table 1 Body composition of CREBH LKO and IKO mice

Body composition of 8-week–old male mice fasted for 24 h and fed was determined by DEXA. n = 7 ~ 10 / group, * $p < 0.05$.

	Fasted		Fed	
	flox	IKO	flox	IKO
Glu (mg/dl)	74.0 ± 3.8	72.6 ± 4.6	161.8 ± 9.3	136.5 ± 5.6*
TG (mg/dl)	232.4 ± 23.2	207.2 ± 15.6	101.5 ± 5.7	105.1 ± 2.9
Cholesterol (mg/dl)	53.8 ± 3.4	64.4 ± 13.6	52.0 ± 3.4	46.2 ± 4.4
FFA (mEq/l)	2.65 ± 0.15	2.39 ± 0.19	1.95 ± 0.13	2.09 ± 0.17
FGF21 (pg/ml)	774.7 ± 154.9	787.0 ± 258.2	88.3 ± 38.6	76.0 ± 27.0

Supplementary Table 2 Plasma parameters in CREBH IKO mice

Samples were collected from 8-week–old male mice fasted for 24 h and fed. n = 7 ~ 10 / group, * $p < 0.05$.

Gene	Fwd primer	Rv primer
Acox1	CGA TCC AGA CTT CCA ACA TGA G	CCA TGG TGG CAC TCT TCT TAA CA
Acta2	CCC AGA CAT CAG GGA GTA ATG G	TCT ATC GGA TAC TTC AGC GTC A
Apoa4	TTA CCC AGC TAA GCA ACA ATG C	GAG GGT ACT GAG CTG CTG AGT GA
Apoa5	GCG AGT TCT GCC GTA GGA C	CCC AAC CCC ATC AAA TGT GA
Apoc2	CCA AGG AGC TTG CCA AAG AC	TGC CTG CGT AAG TGC TCA TG
Apoc3	TAG AGG GCT ACA TGG AAC AAG C	CAG GGA TCT GAA GTG ATT GTC C
Ccl2	CCC ACT CAC CTG CTG CTA CT	ATT TGG TTC CGA TCC AGG TT
Cd68	CCT CCA CCC TCG CCT AGT C	TTG GGT ATA GGA TTC GGA TTT GA
Col1a1	GCT CCT CTT AGG GGC CAC T	ATT GGG GAC CCT TAG GCC AT
Cpt1a	TCT TTC ACT GAG TTC CGA TGG G	ACG CCA GAG ATG CCT TTT CC
Creb3l3	CCA GAC CCT TTA CCC ATA CAT	ATG GTT GGA GGT TAG GGT TCA G
Cyp7a1	GCT GAG AGC TTG AAG CAC AAG A	TTG AGA TGC CCA GAG GAT CAC
Cyclophillin	TGG CTC ACA GTT CTT CAT AAC CA	ATG ACA TCC TTC AGT GGC TTG TC
Elovl2	ACG CTG GTC ATC CTG TTC TT	GCC ACA ATT AAG TGG GCT TT
Elovl5	ATG GAA CAT TTC GAT GCG TCA	GTC CCA GCC ATA CAA TGA GTA AG
Elovl6	ACA ATG GAC CTG TCA GCA AA	GTA CCA GTG CAG GAA GAT CAG T
F4/80	CTT TGG CTA TGG GCT TCC AGT C	GCA AGG AGG ACA GAG TTT ATC GTG
Fasn	ATC CTG GAA CGA CGA GAA CAC GAT CT	AGA GAC GTG TCA TCC TGG ACT T
Fgf21	AGA TCA GGG AGG ATG GAA CA	TCA AAG TGA GGC GAT CCA TA
Fxr	CTC TGC TCA CAG CGA TCG TC	CAC CGC CTC TCT GTC CTT GA
Hmgcr	GAG AAG AAG CCT GCT GCA TA	CGT CAA CCA TAG CTT CCG TAG TT
Hmgcs1	GTG GCA CCG GAT GTC TTT G	CTC TGA CCA GAT ACC ACG TTC
Il1b	GCA ACT GTT CCT GAA CTC AAC T	ATC TTT TGG GGT CCG TCA ACT
Il6	TAG TCC TTC CTA CCC CAA TTT CC	TTG GTC CTT AGC CAC TCC TTC
Ldlr	TGG AGG ATG AGA ACC GGC T	GCA CTG AAA ATG GCT TCG TTT A
Ppara	CCT CAG GGT ACC ACT ACG GAG T	GCC GAA TAG TTC GCC GAA
Scd1	AGA TCT CCA GTT CTT ACA CGA CCA C	CTT TCA TTT CAG GAC GGA TGT CT
Shp	CAA GGA GTA TGC GTA CCT GAA G	CCT GGC ACA TCT GGG TTG AAG
Srebf1a	AGG CGG CTC TGG AAC AGA	TCA AAA CCG CTG TGT CCA GTT
Srebf1c	CGG CGC GGA AGC TGT	TGC AAT CCA TGG CTC CGT
Srebf2	CTG CAG CCT CAA GTG CAA AG	CAG TGT GCC ATT GGC TGT CT
Tgfb1	CCA CCT GCA AGA CCA TCG AC	CTG GCG AGC CTT AGT TTG GAC
Tnfa	TCG TAG CAA ACC ACC AAG TG	AGA TAG CAA ATC GGC TGA CG

Supplementary Table 3 QPCR primer sequences
